# Supplementary material for: Generation of Herbicide-Resistant Soybean by Base Editing
Source: Biology (Basel). 2023 May 19;12(5):741. doi: 10.3390/biology12050741 (PMC10215408; doi:10.3390/biology12050741)
Supplement: Supplementary file 1 [file biology-12-00741-s001.zip › biology-2379368-supplementary.pdf]

Table S1 Primers used in this study.

Name	Sequence (5´- 3´)
Primers for construction of vectors and identification of transgenic plants	
GmAHAS2-P182-F	ATTGAGGTCCCCCGGCGCATGAT
GmAHAS2-P182-R	AAACATCATGCGCCGGGGGACCT
GmAHAS3-P172-F	ATTGAGGTCCCCCGCCGCATGAT
GmAHAS3-P172-R	AAACATCATGCGGCGGGGGACCT
U6-26p-F	TGTCCCAGGATTAGAATGATTAGGC
U6-26t-R	CCCCAGAAATTGAACGCCGAAGAAC
Cas9-F	GAGAGAAGGTCCTCCCAAAGC
Cas9-R	GACCTTATCGTCGAACAGATGC
Bar-F	GCACCATCGTCAACCACTACATC
Bar-R	ACTGCCAGAAACCCACGTCAT
Primers for amplification of the target sequences for sequencing	
GmAHAS1-P178-DF	ACAAGGAAGCGCCGACCAC
GmAHAS1-P178-DR	CCGCAACGGGAATACCAGTG
GmAHAS2-P182-DF	CTGCAGCGCCGGCAACAT
GmAHAS2-P182-DR	CGGCGAGTTGCTGTTGGACG
GmAHAS3-P172-DF	GATCCCTTTGTGCGTTTCGC
GmAHAS3-P172-DR	GGGGCTTAGGCAACCTAGACG
GmAHAS4-P180-DF	CCTCCCTCCCAAACAGTTCCT
GmAHAS4-P180-DR	TCCCTTAGCATAAACAGTCCCG
Glyma02g06360-F	CTTCACAATGCTCCTAATCCA
Glyma02g06360-R	CAACACCAACCCTAGACCC
Glyma07g02610-F	GGGCTGAAATTGCAGACGC
Glyma07g02610-R	CCGCCAGTCAGGTAGTAGCAGT
Glyma07g05680-F	GGTTCAAGGACAGACCAGGAT
Glyma07g05680-R	CCTAACATTGTGACCCCTAACC
Glyma11g31450-F	GGGGTAGACCTGGAGGATTATGT
Glyma11g31450-R	TCCCACTATACCACCCTCTTCTTT
Glyma14g11260-F	CAGCAGAGCACCTCCCAAGA
Glyma14g11260-R	GCAAACAACGCCAGACACG
Glyma15g03350-F	GCGTGCTCATTCGGAGATTC
Glyma15g03350-R	CGCTCCCACAGCATTAGACG
Glyma15g40520-F	CAGCCCCGAGACAGTATCCAA
Glyma15g40520-R	AGCTTCAAATGCAGCGACCTC
Primers for identification of GmAHAS4 P180S homozygous and heterozygous mutants	
GmAHAS4-P180-F	CCTCCCTCCCAAACAGTTCCT
GmAHAS4-180P-R	TGCCGATCATGCGGCGGGGG
GmAHAS4-180S-R	TGCCGATCATGCGGCGGGAA
18S RNA-F	CCTTGCTTGTTGCTTTACTAAATAG
18S RNA-R	ATGCACCTTTTCGTTTGTTTCGGAG

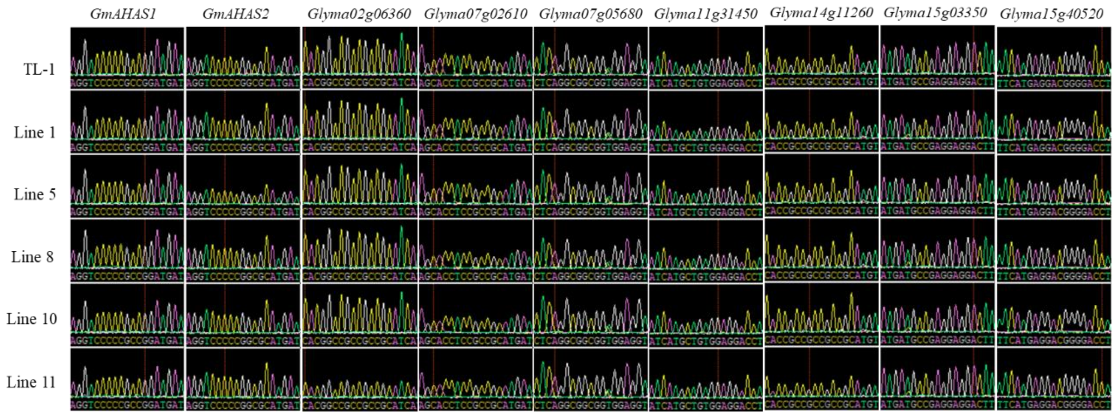

Figure S1 Off-target detection of 5 T0 mutants.

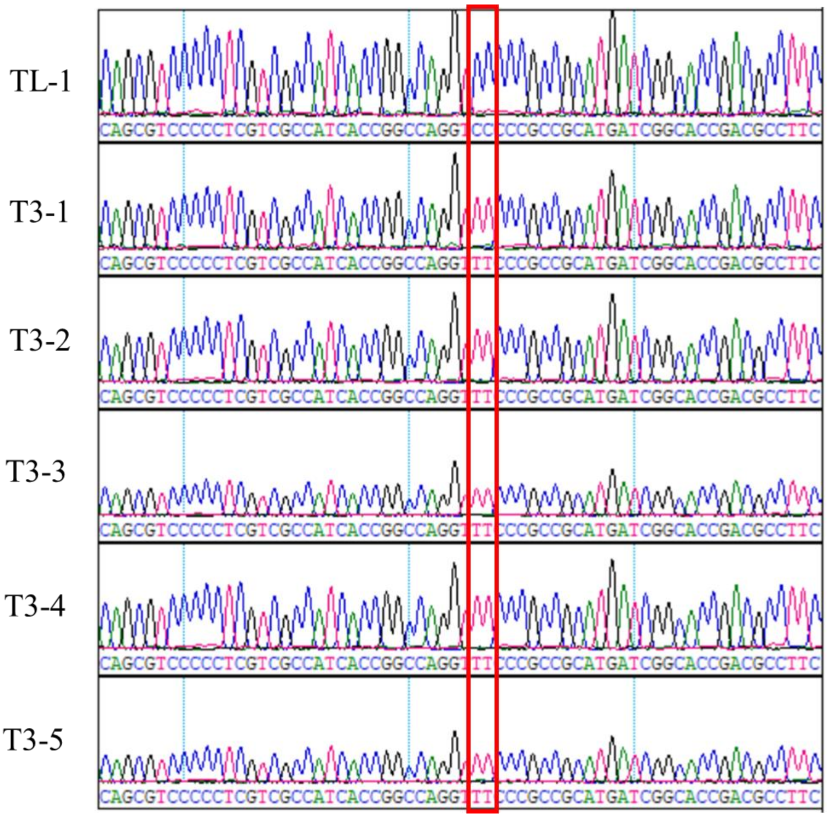

Figure S2 Chromatograms of Sanger sequencing results of T3 generation of GmAHAS4 P180S plants.

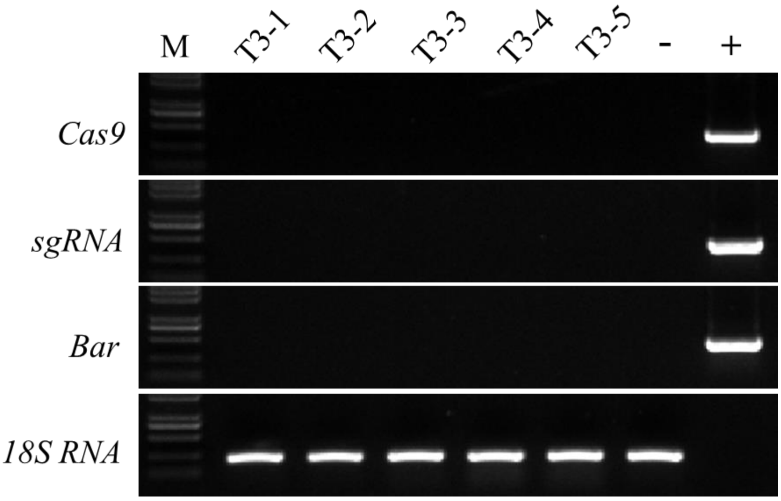

Figure S3 PCR identification T3 generation of transgene-free GmAHAS4 P180S plants.

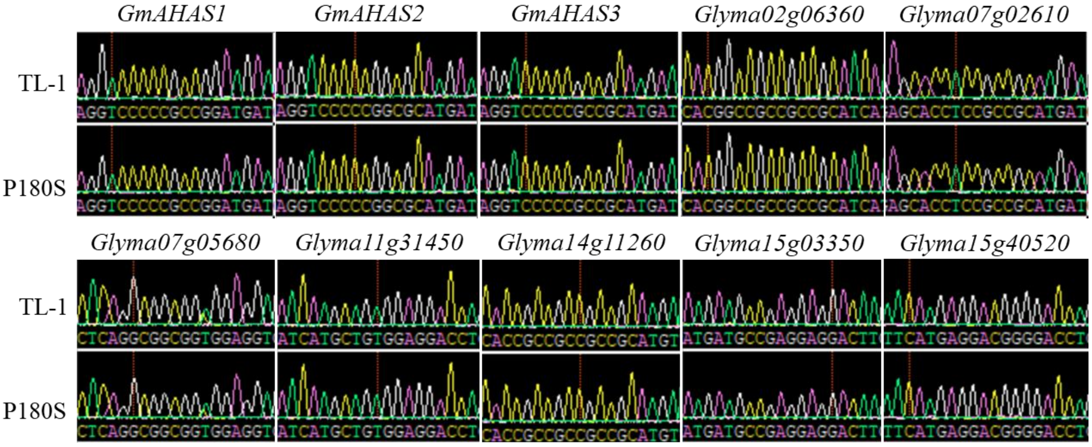

Figure S4 Off-target detection of T3 GmAHAS4 P180S mutants.
